# An effective antimicrobial complex of nanoscale β-cyclodextrin and ciprofloxacin conjugated to a cell adhesive dipeptide†

**DOI:** 10.1039/d2ra05822g

**Published:** 2022-12-15

**Authors:** Reza Taheri-Ledari, Farinaz Jalali, Leili Heidari, Fatemeh Ganjali, Fereshteh Rasouli Asl, Simindokht Zarei-Shokat, Mohadeseh Forouzandeh-Malati, Adibeh Mohammadi, Ali Maleki

**Affiliations:** Catalysts and Organic Synthesis Research Laboratory, Department of Chemistry, Iran University of Science and Technology Tehran 16846-13114 Iran maleki@iust.ac.ir; Department of Chemistry, Faculty of Basic Sciences, Ilam University P. O. Box 69315-516 Ilam Iran

## Abstract

Today, various drug delivery systems (DDS) are utilized to carry and deliver the desired drugs to the targeted action area to reduce potential side effects and negative interactions. Nanomaterials are an excellent candidate for the delivery of potent drugs, as they enhance pharmacokinetic and pharmacodynamic properties. Herein, we present a new ciprofloxacin (CPFX) delivery system based on a polymeric nanocarrier (β-cyclodextrin) conjugated to a cell-adhesive dipeptide structure. Cyclodextrin (CD) is an inexpensive, easily accessible, biodegradable, and biocompatible material. Also, the conjugation of cysteine–arginine (CR) dipeptide to the CPFX/β-CD particles is carried out to enhance cell adhesion growth. Through accurate analysis, the drug content and release for a final product have been estimated to be *ca.* 32%. Overall, the antimicrobial effects of CPFX were considerably raised through a low dose of CPFX. The growth zone inhibition of CPFX/β-CD–CR particles on the *staphylococcus aureus* and the *Escherichia coli* bacterial cells was 5.5 ± 0.2 cm and 3.5 ± 0.2 cm, respectively. Hence, this therapeutic nano bioconjugate is an excellent candidate to be applied in antimicrobial applications with the minimum incorporated CPFX.

## Abstract 2

A soft-matter based antimicrobial nano-cargo made of beta-cyclodextrin and ciprofloxacin is reported.

## Graphical abstract

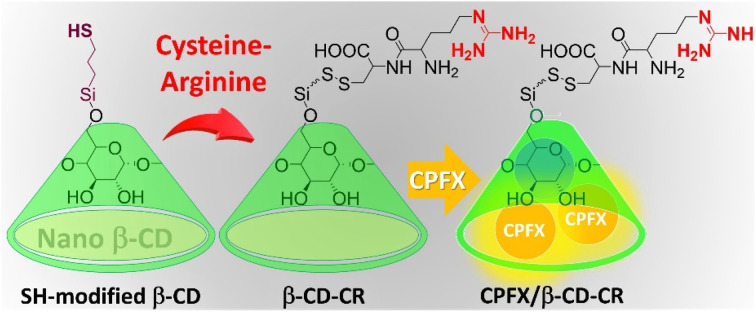

## 1. Introduction

Antibiotics are regarded as the most significant developments in the modern era of medicine because of their tremendous effect on the reduction of mortality in humankind as well as economic harm. Nowadays, antimicrobial resistance (AMR) is the most critical concern in the fight against infectious diseases caused by drug abuse.^1–3^ Indeed, the mechanism of antibiotics action illustrates that antibiotics prevent the synthesis of the bacteria cell walls, disturb the cellular membrane, forbid the synthesis of nucleic acid,^4^ inhibit protein synthesis, block pathways and disrupt metabolism.^5^ Novel quinolones as antibiotics have been extended with enhanced effectiveness and spectrum over the years.^6^ CPFX is a second-generation fluoroquinolone used to treat various infections, including urinary infections, infections related to bone, cystic fibrosis, chronic otitis media, prostatitis, and so on.^7,8^ CPFX demonstrates more remarkable activity against Gram-negative bacteria (Enterobacteriaceae like *Escherichia coli*) than Gram-positive bacteria (*Staphylococcus aureus*).^9^ To clarify the antibacterial mechanism of the CPFX, it is crucial to know that CPFX is a DNA gyrase inhibitor. DNA gyrase is a tetramer consisting of two subunits GyrA and two GyrB subunits. DNA gyrase belongs to a group of enzymes called DNA topoisomerases, which is an essential part of the DNA replication system, and its function is to break the backbone of DNA. The GyrA subunit binds to DNA and hydrolyzes the backbone, while GyrB catalyzes the hydrolysis of ATP, which drives the supercoiling process. Topoisomerase IV is involved in resolving daughter chromosomes after DNA replication as well as the unscrewing of supercoils in the DNA. CPFX do not play a role in the breaking reaction of DNA strands catalyzed by these enzymes but inhibit the recombination step. CPFX are commonly bacteriostatic. However, high doses of CPFX can lead to double-stranded DNA breaks that are lethal to the affected cells.^10^

In fact, one of the best ways to reduce interactions referred to drugs, diminish side effects, and increase the ability to kill bacteria is using appropriate DDS.^11^ Drug delivery means the delivery of pharmaceuticals to a target tissue to achieve safer and better therapeutic outcomes.^12^ DDS has some advantages and disadvantages, such as extending the duration of the action, reducing the frequency of dosage and ensuring the bioavailability of medicines, keeping the drug against degradation, minimize adverse drugs side effects, enhancement the compliance of patients and increase medication use through declining fluctuation plasma concentration. However, the disadvantages include the toxicity of the drug delivery device, the potential for hazardous degradation products, the need for surgery either on applying systems or removal, patients might be uncomfortable using the DDS device, and the cost and usage of DDS may be expensive.^11^

Nanotechnology affects everything from nanoscale gadgets to drug delivery frameworks.^13–15^ Drug delivery to the pain site can be active or passive *via* nanocarriers. In active targeting, peptides and antibodies are conjugated to the DDS *via* targeted tissue receptors, lipids, or antigens. The conduction of drugs *via* the self-assembled nanostructured and released drug encapsulation at the object is related to passive targeting.^16–18^ In the last few decades, the application of nanomaterials in a variety of scientific domains, including; catalysis,^19–27^ photocatalysis,^28,29^ gas adsorption,^30^ applications in environments,^31^ devices in photovoltaic and photoelectric,^32,33^ and drug delivery has grown. There are different types of nanocarriers like; polymeric nanoparticles (NPs), magnetic nanoparticles (MNPs), gold nanoparticles (AuNPs), and mesoporous silica nanoparticles (MSNPs).^34^ Preparing nanocarriers is crucial since many novel drugs have low solubility in water and low bioavailability.^35^ Drug delivery has had a lot of development over the last few decades. As proven, biodegradable and bioabsorbable polymers provide a magical option for many novel DDS. For instance, CD derivatives are one of the natural polymers for polymeric drug delivery carriers.^36^ CD-based nanocarriers can enhance the bioavailability, correct the metabolism of medication, toxicity decline, also boost the drug's half-life.^37^ β-CD has a versatile potential with its suitable geometry for forming inclusion complexes with innumerable drugs.^38^ Furthermore, β-CD is a good option for drug delivery because the size of the cavity (inner cavity diameter: 6.0–6.4 Å, outer diameter: 15.4 Å) is affordable, and the drug loading mechanism improves the bioavailability solubility and stability of cargo.^39^ However, to enhance the accomplishment of these DDSs, nanocarriers may be fitted with attached proteins or chains of synthetic peptides.^38^ Among various species of proteins, peptides do not damage the integrity of the cellular membrane.^40^ The attendance of the specific amino acids in the formation of peptides, such as arginine, is crucial because of its cationic guanidine groups, which are able to interact with anionic sulfate or phosphate groups over the cell membrane.^41^

The study represents an efficient DDS construct from β-CD nano carriers covered by 3-mercaptopropyl trimethoxy silane (MPTMS) cross-linker to deliver the antibiotic CPFX. Additionally, a new dipeptide structure, which has been synthesized in a solid phase, has been employed as an influential factor for penetrating the bacterial cells and better conjugating the structure. Notably, incorporating the peptides into the structure led to the CPFX dosage reduction, even in connection with Gram-negative bacteria. Briefly, the zone of inhibition (ZOI), minimum inhibitory concentration/minimum bactericidal concentration (MIC/MBC), and colony count experiments were accomplished to evaluate the *in vitro* cellular experiments of CPFX/β-CD–CR on Gram-negative (*E. coli*) and Gram-positive (*S. aureus*) bacteria. Nanocarrier integration with the drug content of 32% has enhanced the antibacterial activity of the prepared nanocarrier in comparison with the individual analogous dosage of CPFX. Notably, the derivatives of CD can use as anti-infective agents. Many CDs and their derivatives have low toxicity and resistance to enzymatic breakdown in biological fluids with GRAS (generally regarded as safe) status from the FDA. Moreover, we introduced the bacterial cellular uptake of CPFX by conjugating a dipeptide to CPFX/β-CD and its controlled release in different conditions.

## 2. Results and discussion

### 2.1. Preparation of CPFX/β-CD–CR nanocomplex

**Scheme 1:**
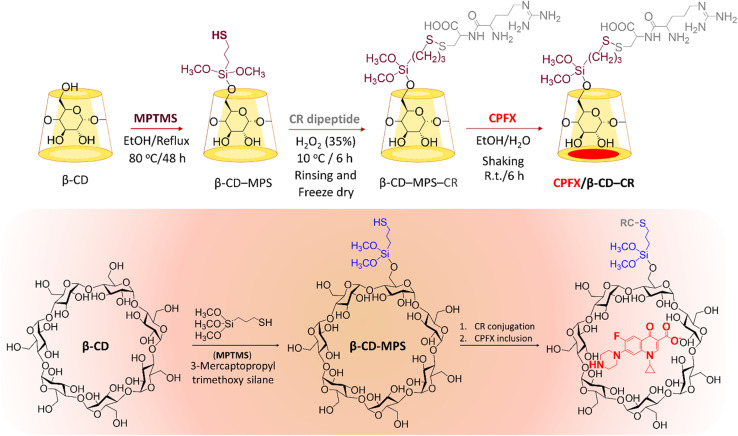
Schematic preparation of CPFX/β-CD–CR nanocomplex. Note: the “MPS” has been eliminated form final formulation (CPFX/β-CD–MPS–CR), as it does not play any significant role in the application.

In the first stage, the surface of β-CD was modified with MPTMS to introduce –SH groups onto the surface. In the third stage, the synthesized CR dipeptide is connected to the surfaces *via* the foundation of a disulfide bond (Scheme 1). The dipeptide is created from 2-chlorotrityl chloride (CTC) resin through solid-phase synthesis.^44^ In this regard, the resin was washed with dimethyl formamide (DMF) and dichloromethane (DCM) to provide a pure and inflated CTC complex. Following this, cysteine amino acid and *N*,*N*-diisopropylethylamine (DIEA) were mixed in the DMF, and all contents were shaken at room temperature (25 °C). Then, the capping step was completed *via* methanol solution; subsequently, the protection group of Fmoc was eliminated with a piperidine solution in the DMF (25%). Then a blend of arginine amino acid, DIEA, and *N*,*N*,*N*′,*N*′-tetramethyl-*O*-(benzotriazole-1-yl)-uronium tetrafluoroborate (HBTU) has been appended to the system and shaken. Finally, the last deprotection was performed, and the CTC was separated *via* trifluoroacetic acid (TFA) (95%) from the complex. The structure of the synthesized CR dipeptide was confirmed by H-NMR spectroscopy (Fig. S1, in the ESI section†). To conjugate the CR dipeptide with the surface of the CPFX, a disulfide bond has been constructed between the mercaptopropyl silane (MPS) and the cysteine amino acid thiol end groups. In this step, β-CD–MPS particulates have been dispersed into distilled water through an ultrasonic ice bath. Next, dropwise ethanol was added to a mixture of CR dipeptide solution with partial quantities of hydrogen peroxide. The CPFX drug was loaded into the functionalized nanocarrier in the next stage. The inner cavity of β-CD is lipophilic, whiles the outer one is hydrophilic, which makes their complex formulation possible with lipophilic molecules. The β-CD can encapsulate molecules inside their cavity *via* non-covalent interactions such as electrostatic, van der Waals, hydrophobic, and hydrogen bonds to form an inclusion complex of host–guest type.^42,43^ Moreover, computational modeling has shown that van der Waals forces are the most important driving forces for the complexation and the moiety (piperazinyl) of the CPFX drug structure, which is included in the internal cavity of β-CD.^45^ Consequently, through centrifugation, the CPFX/β-CD–CR particulates were collected.^46^ In order to verify the prepared structures within the preparation route (presented in Scheme 1), H-NMR spectra of the neat β-CD, β-CD–MPS, and CPFX-included β-CD–MPS were prepared, as well. However, for evaluation of the CPFX inclusion (and also CR conjugation) into the structure, the H-NMR spectrum of CPFX/β-CD–CR was provided, but it was crowded and not quite interpretable. In fact, the signals in the same chemical shift areas extensively overlapped in the H-NMR spectrum. Therefore, the H-NMR spectrum of “CPFX/βCD–MPS” complex as a clue on the preparation of CPFX/βCD–CR complex was provided, as it is less crowded and the signals related to the included CPFX are quite interpretable. However, the H-NMR spectrum of CPFX/β-CD–CR has also confirmed the presence of CPFX into the structure, where the signals related to the aromatic protons of CPFX have appeared between 6.0 and 8.0 ppm. Also, the observed integrated amounts of the protons in the mentioned area can be another verification for the CPFX inclusion. As exhibited in Fig. S2–S8 (in the ESI section†), all of the shown structures in the scheme are corroborated by the NMR data.

### 2.2. Characterization of CPFX/β-CD–CR nanocomplex

#### 2.2.1. Fourier-transform infrared spectroscopy

**Fig. 1:**
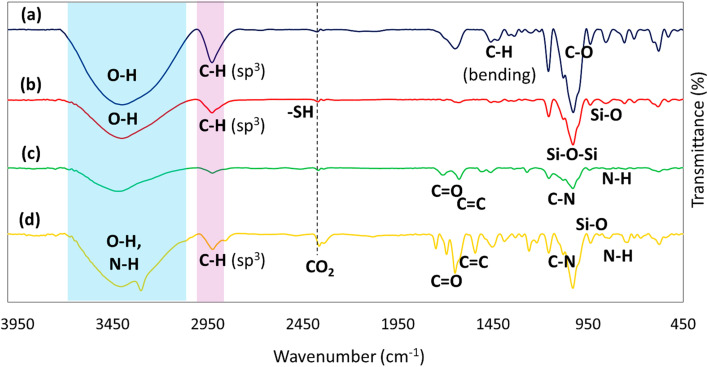
FTIR spectra of (a) β-CD, (b) β-CD–MPS, (c) β-CD–MPS–CR, (d) CPFX/β-CD–CR nanocomplex.

The Fourier-transform infrared (FTIR) spectroscopy is a method used to acquire the infrared spectrum of absorption, emission, and photoconductivity of solid, liquid, and gas. It is utilized to distinguish various functional groups in the materials. As demonstrated in Fig. 1a, the broad pick at 3400 cm^−1^ is related to the O–H bonds, and the peak of the C–H bond with hybridation sp^3^ appeared at 2900 cm^−1^, stretching vibrations. As well as this, the peak of the bending vibrations of the C–H bond with hybridation sp^3^ emerged at 1400 cm^−1^, and peaks at 1100 cm^−1^ showed C–O stretching.^47^ In Fig. 1b, a sharp peak at 1100 cm^−1^ and a peak at 800 cm^−1^ are allocated to Si–O–Si and Si–O stretching vibrations, respectively. There is also a weak peak to illustrate the position of the mercapto (–SH) group at the absorption peak of 2500 cm^−1^.^48^ Additionally, in Fig. 1c, another broad peak in the region of 3400 cm^−1^ is represented by the N–H stretching group. In Fig. 1d, the band at 1650 cm^−1^ exhibited C=O (carbonyl) and C=N stretching.^49^ Among the vibrations, those at 1100 cm^−1^ and 700 cm^−1^ are assigned to the vibration absorption of C–N stretching and secondary amine, respectively.^50^

#### 2.2.2. Energy-dispersive X-ray spectroscopy

**Fig. 2:**
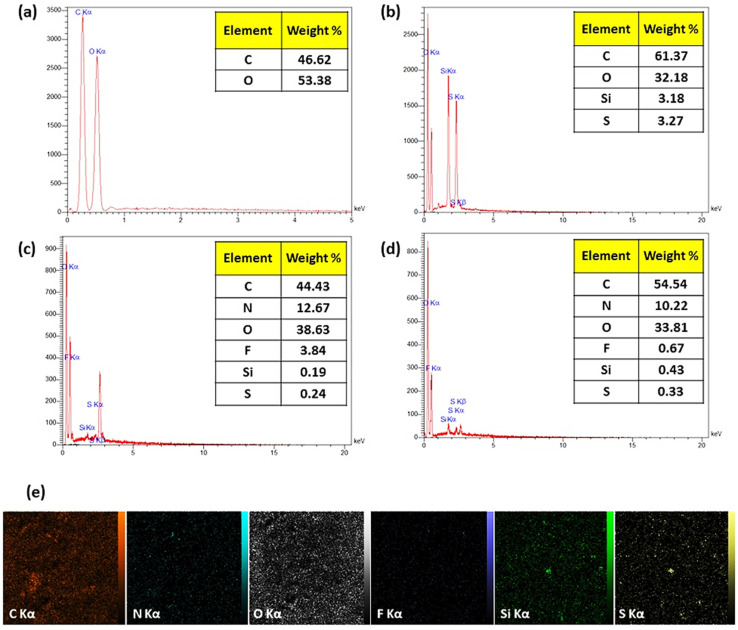
EDX spectra and quantitative results of (a) β-CD, (b) β-CD–MPS, (c) β-CD–MPS–CR, (d) CPFX/β-CD–CR, (e) EDX mapping of the CPFX/β-CD–CR nanocomplex.

Energy-dispersive X-ray spectroscopy (EDX) is based on the production of characteristic X-rays, highlighting the identity of the elements within the sample (Fig. 2). The attendance of O and C atoms has been verified in each of the specimens *via* the emerged signals at 0.5 and 1.8 keV, respectively. The existence of MPS was checked through a signal revealed at 2.3 and 1.7 keV (spectrum b), which is related to S and Si atoms, respectively. The only released electrons from the F atom (3.84% of the total weight) are referred to as CPFX (spectrum c). The peak intensity associated with carbon and sulfur components is gained by conjugating the CR dipeptide (spectrum d).

#### 2.2.3. Thermogravimetric analysis

**Fig. 3:**
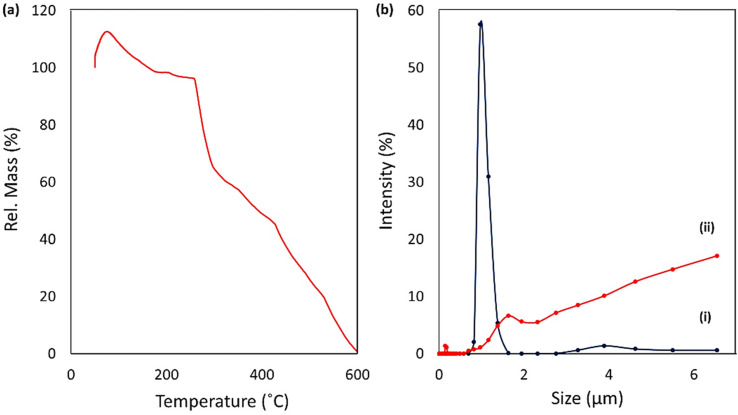
(a) TGA curve of CPFX/β-CD–CR, and (b) intensity-based DLS curves of particle size (zeta-average) and size distribution of (i) β-CD–CR and (ii) CPFX/β-CD–CR nanocomplex.

The thermal degradation state of the prepared CPFX/β-CD–CR nano-cargo was investigated by thermogravimetric analysis (TGA). Since the whole structure is made of a hydrocarbon skeleton, it was expected to see a rapid and complete degradation trend over the heating process.^51^ As presented in Fig. 3a, the thermal degradation is initiated from 75 °C with a rel. mass value of *ca.* 113%, and continued to 600 °C. At first glance, this is assumed that an error has occurred in the data due to the excess 13 wt% at the beginning, but it is a typical occurrence in the polymeric hydrogel structures that a large volume of the moisture in the air is adsorbed by a partial increase in the temperature.^52^ In the whole trend of degradation, this is observed that a 17% weight loss (113–96%) has occurred *via* heating the sample up to *ca.* 260 °C, due to the quick elimination of water molecules from surfaces and the underlying layers of CPFX/β-CD–CR nano-cargo.^53^ At the following stage, *ca.* 32% of the total weight (96–64%) disappeared in a thermal range of 260–290 °C. This fast drop in weight can be ascribed to the removal and degradation of the CPFX drug encapsulated into the polymeric β-CD strands.^54^ Afterward, degradation of the β-CD–CR structure started from 300 °C and continued to *ca.* 600 °C, where the whole structure was mainly decomposed to CO_2_ molecules. These results can essentially confirm the structural attributes of the CPFX/β-CD–CR nanocomplex.

#### 2.2.4. Zetasizer analysis

This method determined particle size, distribution, intensity-based dynamic light scattering (DLS), and zeta potentials of NPs were determined. DLS prepares a particle size measurement from scattered light within the solution. This analysis was performed in the stage of CPFX/β-CD, and that of CPFX/β-CD–CR, and the size of the particles was investigated (Fig. 3b). According to the obtained results, it is deduced that when the drug (CPFX) is loaded into the modified polymer structure (β-CD), the size of 96% of the particles is about 210 nm. The rest of the material is around 2 μm (blue curve). Also, the polydispersity index (PDI) factor was 4.69, demonstrating particle accumulation. The 2.0 μm size of particles may be due to the agglomeration of polymer material. In the next stage, and after conjugating the synthesized dipeptide to the structure, the analysis was also done to check the particle size. It was seen that 77% of the particles are approximately 3.0 μm and 20% of them are 600 nm (PDI = 2.93). It is likely that because of the agglomeration and hydrogen bond between dipeptide and polysaccharide structure, the size of the particles has grown larger. Zeta potential analysis is a crucial characterization approach to appraise the surface charge. Zeta potential (positive or negative) can influence the stability of particles and their cellular adhesion. The surface charge is caused by the physical interaction between the surface and particles. In this project, before conjugating the dipeptide, the ZETA potential was 13.7 mV; however, after the dipeptide conjugation, this amount increased to 14.7 mV.^55^

#### 2.2.5. XRD patterns

**Fig. 4:**
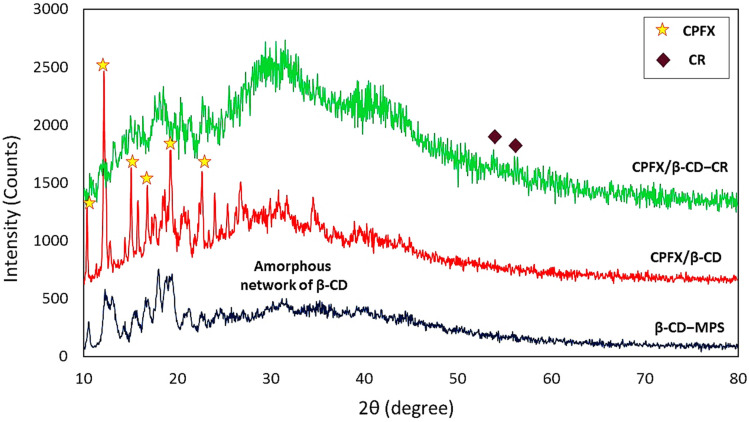
XRD analysis of the β-CD–MPS, CPFX/β-CD–MPS, CPFX/β-CD–CR nanocomplex.

X-ray powder diffraction spectroscopy can identify a complex's active ingredients and crystalline excipients. Fig. 4 shows that the complex does not have observable crystallization in the XRD results. The X-ray diffractogram of β-CD indicated peaks in the range of 10–15° and 15–20° (2*θ*) as well as the significant peaks at 2*θ* ≈ 12°, 18°, and 19°, which cause confirmation of the amorphous features of β-CD in nature.^56^ Moreover, the CPFX influences the morphology of the previous structure, and a rise in peak intensity is observed referring to pure CPFX that shows the sharp peaks at 2*θ* = 14.4°, 20.7°, 25.5° as well as the weak peaks at 2*θ* = 8.3°, 13.4°, 16.5°.^57^ As can be seen, some new weak peaks in the pattern of CPFX/β-CD–CR are attributed to the CR dipeptide on the surfaces.

#### 2.2.6. Electron microscopy imaging

**Fig. 5:**
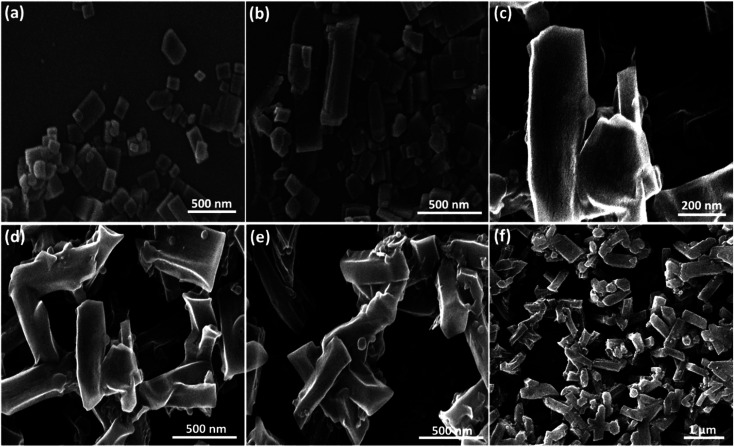
FESEM images of (a) and (b) β-CD, (c) and (d) β-CD–MPS–CR, (e) and (f) CPFX/β-CD–CR nanocomplex.

The field emission scanning electron microscope (FESEM) is utilized to investigate morphology (such as particle size and shape), metallographic details, uniformity, topology, and the investigation of elemental compositions. For the SEM sample preparation, we dispersed each sample in ethanol and sonicated them in a cleaner bath at an ambient temperature. A few amounts of the samples were poured on the glass laminate. As shown in Fig. 5, the size of particles in pure β-CD is about 200 nm. In the case of modification of the polymer strands by MPS and further conjugation to CR dipeptide structure, the particle size has increased to 500 nm, while the uniformity is still confirmed. The final product demonstrates the rod morphology of particles with an average size of 1.0 μm and high uniformity. The rods have more rapid enrichment and higher susceptibility; they also have the capability for major adhesion because of their high contact area. Rod-shaped particles can easily penetrate cells through the tip first. Nanorods with their main vertical axis tend to incorporate with the cell membrane. The rod-shaped nanoparticles achieved a superior targeting efficiency compared to other nanoparticle shapes like spherical, plate, or flake.^58^ Consequently, based on these data, it can be claimed that the designed nano-size cargo is suitably internalized into the target bacteria cells because the mean size and the general morphology of the particles are preferred in the cellular uptake process by the living cells.^59^

### 2.3. Antimicrobial properties of CPFX/β-CD–CR nanocomplex

The significant advantage of nanocarriers for targeted DDS is increasing the consistency of medicine and optimizing the half-life in blood serum.^60^ The carriers decline side effects disturbance and boost drug resistance against degradation. Moreover, nanocarriers exhibit more therapy advantages with low dosages of drugs, and low doses of the drug delivery to the target cell could be faster and more controlled.^61^ As mentioned in this literature, β-CD is ideal for drug delivery, and the drug loading mechanism improves cargo's bioavailability, solubility, and stability. Also, peptides are regarded as very effective and safe methods that do not damage the integrity of the cellular membrane. They can pass *via* different cell types of membranes. Some amino acids' attendance in forming the peptides, such as arginine, is crucial because it can interact with anionic sulfate or phosphate groups over the cell membrane. The MPTMS is the agent to create a disulfide bond between cysteine amino acids and can increase the antimicrobial effects. This study provided an efficient DDS construct from β-CD NPs covered by an MPTMS cross-linker to deliver the antibiotic CPFX. Furthermore, a new dipeptide chain, which has been synthesized in the solid phase, was utilized as a more efficient factor for entering the bacteria cells and conjugating them to the structure.

#### 2.3.1. CPFX loading in β-CD–CR

**Fig. 6:**
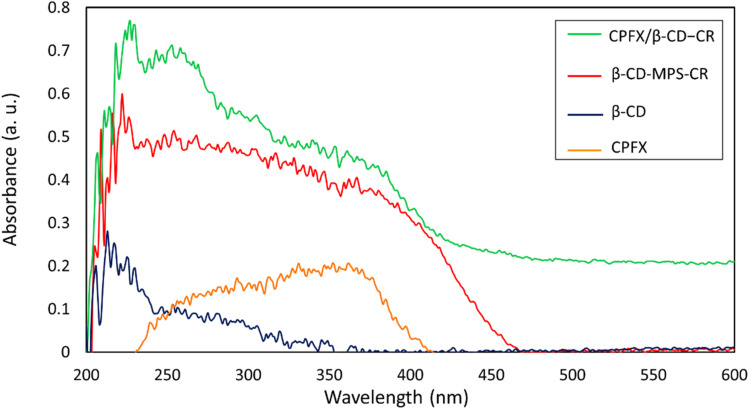
UV-DRS spectra of the neat β-CD, CPFX, β-CD–MPS–CR, and CPFX/β-CD–CR nanocomplex.

Solid-state UV-vis diffuse reflectance spectroscopy (UV-DRS) was utilized to confirm the successful loading of CPFX into the nanocarrier. As seen from the curves of Fig. 6, the β-CD spectrum indicates a UV reflectance activity in a wavelength range of 205–250 nm, including the sharp peaks at 211 and 225 nm. Also, the maximum reflectance by the neat CPFX is situated in a range of 250–400 nm. Indeed, a noticeable reflectance activity for the CR-conjugated β-CD–MPS demonstrates successful attachment of CR onto the surfaces. Consequently, the final product has a considerable absorbance in a long range of 200–450 nm, confirming the CR conjugation and CPFX loading into the structure.

#### 2.3.2. Release of CPFX from CPFX/β-CD–CR and drug content

**Fig. 7:**
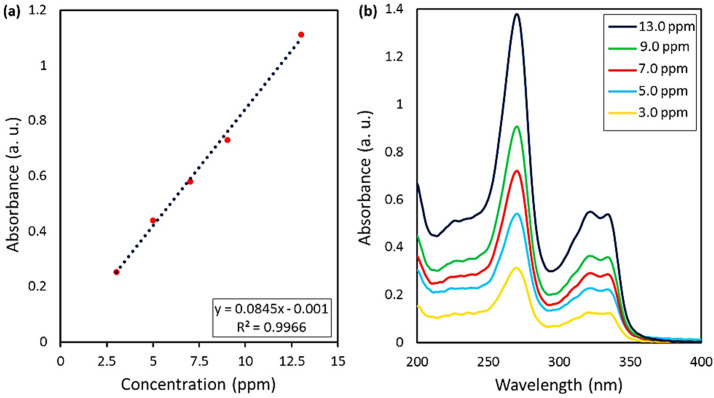
(a) Calibration curve of the standard CPFX solutions, obtained by UV-vis spectrophotometry, (b) UV-vis spectra of CPFX at various concentration values.

The food and drug administration (FDA) approved some approaches to specify the drug loading content (DLC) and drug loading efficiency (DLE), which will be mentioned below. *In vitro* condition, release studies were accomplished in acetate buffer (ACB 0.1 M, pH = 4.6) and phosphate buffer solutions (PBS 0.1 M, pH = 6.8 and 8.0) at 37 °C. Firstly, a calibration curve according to five standard solutions of CPFX (*λ*_max_ = 276 nm) with concentrations of 3, 5, 7, 9, and 13 ppm was acquired (Fig. 7). The CPFX release behavior was measured through UV-vis spectroscopy and compared with the calibration curve at different times (30, 60, 90, 180, 240, 300, and 360 minutes). Initially, the nanoparticles were centrifuged. Then, they were filtered with Whatman filter paper. Also, the blank sample was filtered with Whatman filter paper so as to eliminate the error related to the particulate matter of the paper filter. At last, the solution containing 5.0 mg of CPFX was diluted for a correct UV-vis spectroscopy study. Afterwards, eqn (1) and (2) could be considered for all drug-loading content and release assessments. In the present study, the CPFX loading efficiency in the CPFX/β-CD–CR system was calculated to be 32.0%. According to Beer–Lambert principles, dilution of the sample is necessary to establish an accurate correlation between UV-vis (a.u.) and concentration (ppm). Hence, raw samples need 1/20 dilution with appropriate buffer media. Therefore, in acidic (optimum) conditions, *A* (UV-vis absorbance) was 1.203 (a.u.) for the sample after the drug release process. Afterwards, *X* which is the concentration of CPFX (ppm), and *C* which is the concentration of the CPFX solution (mg mL^−1^) were calculated *via* provided equations (*X* = 301 ppm, *C* = 0.301 mg mL^−1^).^45^

1 *X* = (*A* + 0.001)/(0.0845 × 0.05)

2 *C* = *X* × 1/1000

**Fig. 8:**
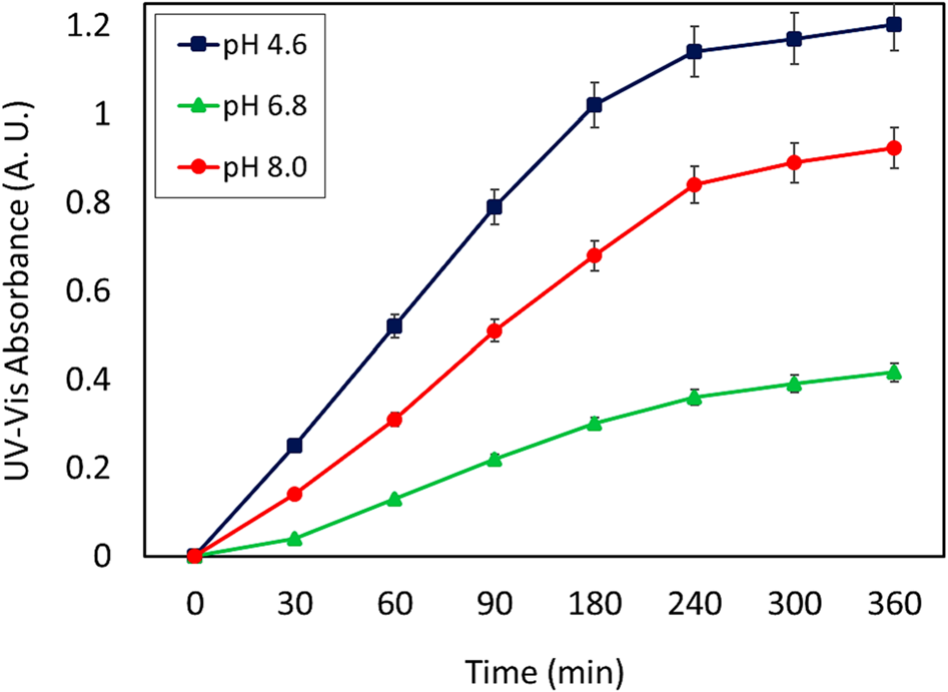
The release profile of CPFX from CPFX/β-CD–CR nano-cargo in three different buffered conditions ACB (0.1 M, pH = 4.6), PBS (0.1 M, pH = 6.8) and PBS (0.1 M, pH = 8.0) [the errors (±) represent the relative error values in each case (*n* = 3)].

As can be seen, the maximum value of the released CPFX has been shown in Table 1, which was regarded as the approximation of the CPFX content (*ca.* 30 ± 0.2) wt% in the CPFX/β-CD–CR system. It can be deduced that inside the bacteria cell (following endocytosis), releasing the encapsulated CPFX has occurred in the acidic condition, which is a foundation of the entire released quantity (100%), and the remaining extents were compared to this measure. As presented in Fig. 8, the maximum drug release of CPFX from CPFX/β-CD–CR nano-cargo occurred when the particles were dispersed in an acidic medium.

**Table 1:** CPFX-release data for CPFX/β-CD–CR nanocomplex under different conditions

Entry	Condition^a^	Absorbance (a.u.)	Concentration (mg mL^−1^)	CPFX release (%)	Rel. E^b^ (%)
1	ACB (0.1 M, pH = 4.6)	1.203	0.301	100^c^	3.5
2	PBS (0.1 M, pH = 6.8)	0.416	0.104	34.55	5.2
3	PBS (0.1 M, pH = 8.0)	0.924	0.231	76.74	4.6

a CPFX release was investigated at 37 °C and 4 h.

b % Rel. E: relative error percentage for three identical samples.

c Complete value of release (100%) was considered for most of the drugs released, and the remaining values were compared to the main value.

#### 2.3.3. Antimicrobial properties: zone of inhibition experiment

To determine the antibacterial characteristics of the CPFX/β-CD–CR therapeutic nano-bioconjugate, the growth rate of two bacterial strains, for example, *S. aureus* and *E. coli*, was investigated according to the presence of the control *via* the ZOI test.^62,63^*S. aureus* and *E. coli* were selected as Gram-positive and Gram-negative bacteria with different surface characteristics, respectively. Features such as cell adhesion to solid surfaces and flocculation strongly depend on surface properties, surface electrical connections, and surface hydrophobicity. The outer cell membrane of Gram-negative bacteria, including *Escherichia coli* (*E. coli*), is covered with a lipopolysaccharide layer of 1–3 pm thickness. Whereas the surface of Gram-positive bacteria such as *Staphylococcus aureus* (*S. aureus*) has a peptidoglycan layer that teichuronic acid, teichoic acid, and proteins are covalently attached to it. For this reason, there is a significant difference in the electrophoretic behavior of the two bacteria, which leads to a significant difference in the surface properties between the two types of bacteria. So, they were selected as two common Gram-positive and Gram-negative bacteria in this research.^64^

**Fig. 9:**
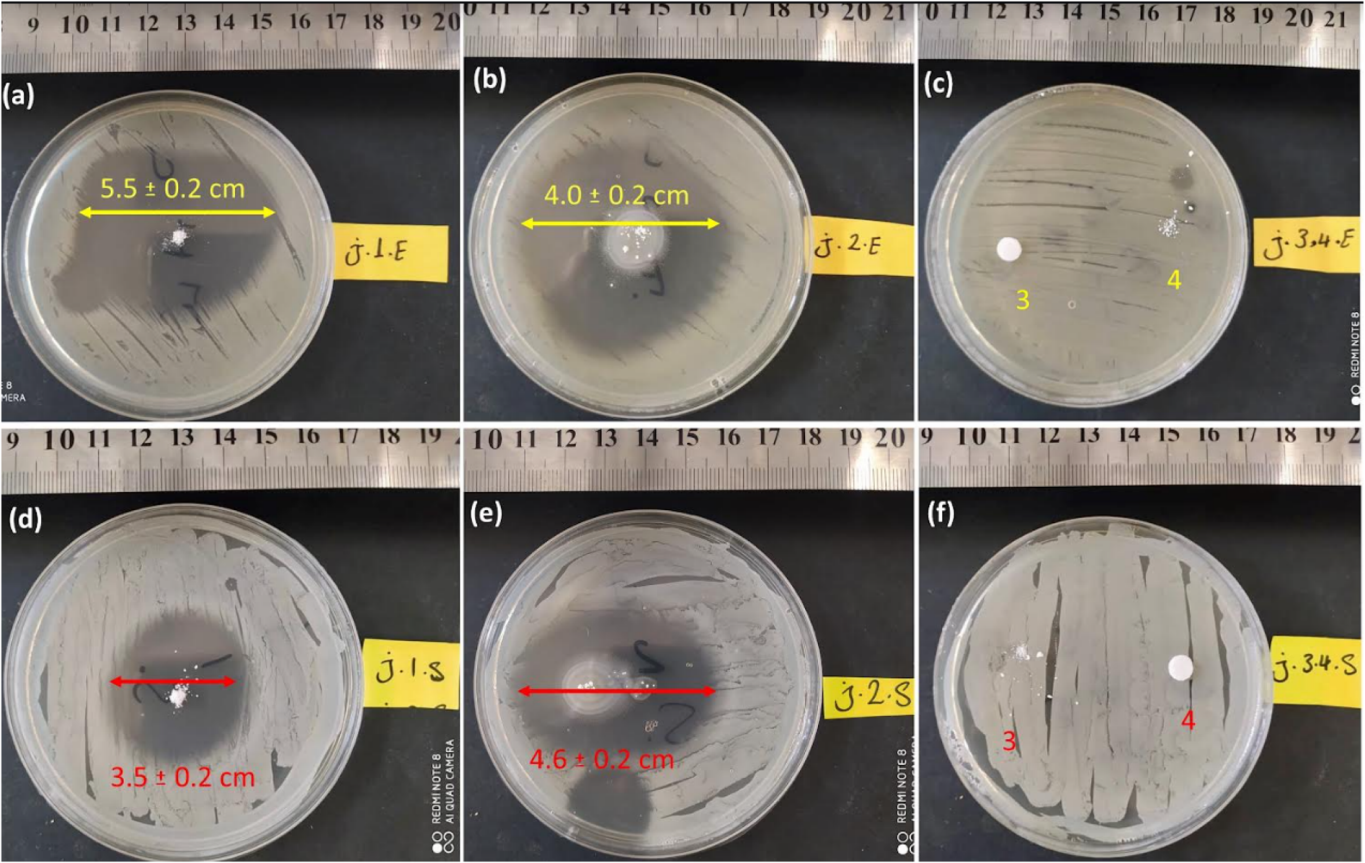
Digital photos of the growth-inhibition zones created by CPFX/β-CD–CR nano-cargo, dipeptide sequence, and individual CPFX (with an equal dosage: 2.0 mg), on *E. coli* (a)–(c) and *S. aureus* (d)–(f) cell lines [errors (±) represent absolute errors per three repeated samples (*n* = 3)]. (a) CPFX/β-CD–CR (nano-cargo), (b) CPFX (drug), (c) β-CD (3), CR (4) on *E. coli*, (d) CPFX/β-CD–CR, (e) CPFX, (f) β-CD (3), CR (4) on *S. aureus* strain.

This assay tested similar dosages of CPFX/β-CD–CR, β-CD, CR, and CPFX. The powdery water-undissolved samples were situated on the agar plates containing bacterial cells. In this regard, 2.0 mg of powder samples were transformed onto agar gels containing *S. aureus* and *E. coli*. The disks were incubated at 37 °C, with a humidity of 95% for 24 h. After incubation, the inhibition zone diameter was measured. Fig. 9a displays that the ZOI of *E. coli* was larger than *S. aureus*, which is attributed to the CPFX/β-CD–CR therapeutic nano-bioconjugate. The growth zone inhibitory of CPFX/β-CD–CR particles on the *E. coli* and the *S. aureus* bacterial cells was 5.5 ± 0.2 cm and 3.5 ± 0.2 cm, respectively.

In contrast, as shown in Fig. 9b, the ZOI measurements for the individual CPFX on the *E. coli* were reduced to 4.0 ± 0.2 cm. Concludingly, the CPFX/β-CD–CR therapeutic nano-bioconjugate with about 30% drug content has higher antibacterial activity than the individual CPFX with the same dosage. Significantly, due to the thicker, strong walls of Gram-negative *E. coli* bacteria, CPFX hardly penetrates the membrane; hence, the ZOI of the *E. coli* samples is larger than in the case of *S. aureus*. In addition, the dipeptide sequence and polymer effects on the ZOI test have been evaluated. In this respect, the individual dipeptide and polymer did not demonstrate the ZOI onto *S. aureus* and *E. coli* cell lines, indicating no antibacterial activity of the dipeptide and polymer (Fig. 9c and f).

#### 2.3.4. Determination of minimum inhibitory and bactericidal concentrations. The minimum inhibitory concentration (MIC) and the minimum bactericidal concentration (MBC)

**Fig. 10:**
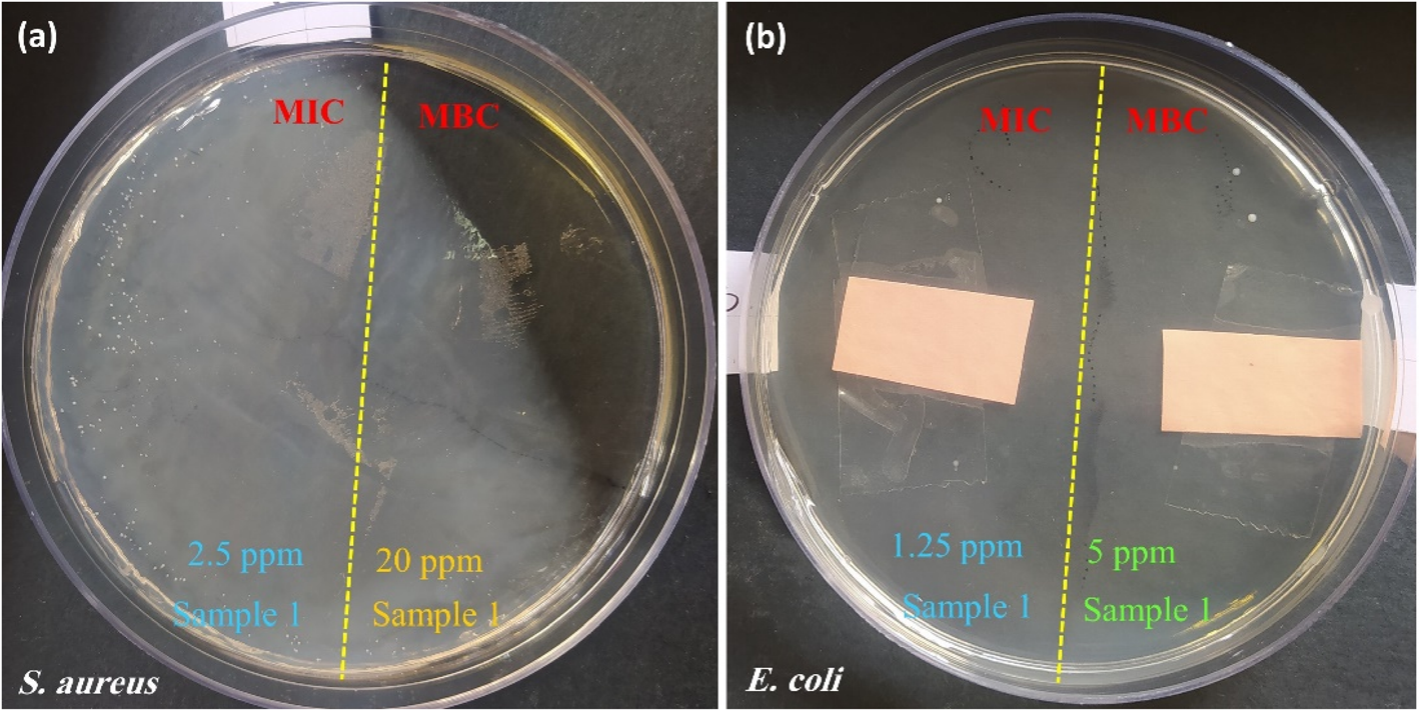
Digital photos of MIC/MBC disks containing (a) CPFX/β-CD–CR nano-cargo in *S. aureus* (MIC = 2.5 μg mL^−1^ and MBC = 20 μg mL^−1^), and (b) CPFX/β-CD–CR nano-cargo (with an equal dosage: 2.0 mg) onto *E. coli* cell lines (MIC = 1.25 μg mL^−1^ and MBC = 5 μg mL^−1^) incubated 48 hours at 37 °C and a humidity of 95%.

Due to the results of the ZOI experiment, the MIC/MBC and colony count approaches were accomplished for CPFX/β-CD–CR therapeutic nano-bioconjugate and individual CPFX on *S. aureus* and *E. coli.* For MIC/MBC experiment, a dilution series was prepared in a sterile test tube.^65^ First, the amount of the CPFX/β-CD–CR particles have been dispersed in deionized water. Afterwards, 10 solutions were made *via* dilution of Mueller–Hinton broth (labeled 1 to 8). The first tube was labeled as C−, indicating CPFX/β-CD–CR control, and the final tube was labeled as C+, showing the growth control. Then, 1.0 mL of Mueller–Hinton broth was injected into each tube, and 1.0 mL of the CPFX/β-CD–CR mixture was poured into tubes No. 1 and C−. Next, 1.0 mL of tube No. 1 contents were conveyed into tube No. 2. After pipetting the contents of tube No. 2, 1.0 mL of its content was moved to tube No. 3, and so on. This process was followed to tube No. 8. The C− labeled tube was applied as CPFX/β-CD–CR control, and the last C+ labeled tube was used as growth control, receiving no antibacterial agents. Next, the microplate was exposed to incubation with 1.0 mL of the culture of *S. aureus* and *E. coli*. Then, the microplate was incubated under similar conditions for 24 h. At last, the microplate was tested for bacterial growth, and the MIC/MBC contents were assessed for the CPFX/β-CD–CR therapeutic nano-bioconjugates (Fig. 10). The MIC/MBC assay data is represented in Table 2.

**Table 2:** The MIC and MBC values for the prepared CPFX/β-CD–CR therapeutic nano bioconjugate and CPFX as a standard

Bacterial strain	Sample 1 (CPFX/β-CD–CR)	Sample 2 (ciprofloxacin)
*S. aureus*	MIC (μg mL^−1^): 2.5	MIC (μg mL^−1^): 0.5
	MBC (μg mL^−1^): 20.0	MBC (μg mL^−1^): 2.0
*E. coli*	MIC (μg mL^−1^): 1.25	MIC (μg mL^−1^): 0.02
	MBC (μg mL^−1^): 5.0	MBC (μg mL^−1^): 0.04

Further assessments on the antibacterial characteristics of the CPFX/β-CD–CR therapeutic nano-bioconjugates were accomplished through the colony count assay.^66^ In this account, the linear cell culture pattern was implemented on the agar gel. Then, the sample was spread throughout the disks and incubated at 37 °C, with 95% humidity for 48 h. The results revealed that the *S. aureus* population on the CPFX/β-CD–CR therapeutic nano-bioconjugates-containing disk after 30, 60 and 180 minutes was 250.0, 150.0 and 5.0 (×10^8^ CFU mL^−1^), respectively, and *E. coli* bacteria population on the CPFX/β-CD–CR therapeutic nano-bioconjugates-containing disk during those times was 10.0, 5.0 and diminished to zero. Notably, the CPFX/β-CD–CR nano-cargo's cell-killing activity with about 30% drug content was analogous to the individual CPFX with a similar concentration. As expected, no bacterial population difference was seen between the control sample and the CR-containing disk.

## 3. Experimental section

### 3.1. Materials and instruments

All chemicals and devices utilized in this study are listed in Tables 3 and 4.

**Table 3:** The brand and purity of the utilized chemicals in this study

Materials	Brand & purity
Beta cyclodextrin	Sigma-Aldrich
(3-Mercaptopropyl)trimethoxy silane (MPTMS)	Sigma-Aldrich, 97.0%
Ciprofloxacin	Sigma-Aldrich
*N*,*N*,*N*′,*N*′-Tetramethyl-*O*-(benzotriazol-1-yl)uranium tetrafluoroborate (TBTU)	Sigma-Aldrich, ≥97.0%
Fmoc-Arg(Pbf)-OH	Sigma-Aldrich, ≥98.0%
Fmoc-Cys(Trt)-OH	Sigma-Aldrich, ≥95.0%
2-Chlorotrityl chloride (CTC) resin	Sigma-Aldrich, 97.0%
*N*,*N*-Diisopropylethylamine (DIPEA)	Sigma-Aldrich, ≥99.0%
*N*,*N*-Dimethylformamide (DMF)	Sigma-Aldrich, 99.8%
Dichloromethane (DCM)	Sigma-Aldrich, 99.9%
Piperidine	Sigma-Aldrich, ≥99.0%
Trifluoroacetic acid (TFA)	Sigma-Aldrich, ≥99.0%
Synthetic ethanol	Merck, 99%
Synthetic methanol	Merck, 99%

**Table 4:** The brand and model of the used devices in this study

Equipment	Brand
FT-IR spectroscopy	Shimadzu FT-IR-8400S
EDX spectroscopy	VEGA-TESCAN-XMU
TGA analysis	Bahr-STA 504
DLS analysis	Horiba (SZ-100)
XRD	DRON-8 X-ray diffractometer
FESEM	Zeiss Sigma
Solid state UV-vis spectroscopy	Shimadzu-UV-2550/220v
UV-vis spectroscopy	Beckman DU640
Ultrasonic cleaner bath	BANDELIN electronic GmbH & Co. KG
Centrifuge	Beckman Coulter GmbH
Oven	Genlab Ltd
Freeze drier	KASSEL Machinery (Zhejiang) Co., Ltd
Rotary evaporator	Heidolph Instruments GmbH & Co. KG
Incubator	Sh, Noor Sanat Ferdos
Shaker	VWR 5000
Autoclave (for sterilization)	Reyhan Teb, 2KW-220v
Vacuum pump	Heidoph persia LQ1

### 3.2. Preparation methods

#### 3.2.1. Modification of β-CD by MPS

There are some steps to functionalize the structure of the polymer: first, the dissolution of precursor 1.0 g β-CD into 20.0 mL distilled water. Next, 15.0 mL MPTMS was dissolved into the 10.0 mL ethanol, and then the mixture was added to the above precursor solution, followed by subsequent ultrasonic treatment for 10 min. Afterwards, the mixture was conveyed into a 50.0 mL round-bottom flask and refluxed at 80 °C for 48 h under vigorous stirring conditions. Then through the rotary device, the solvents separated, and the collected product was washed with ethanol (10.0 mL × 3) and water (10.0 mL × 3). Finally, the white product was separated from the solution by centrifugation at 9k rpm for 10 min, and the resulting particles were dried for 12 h in an oven at 60 °C.

#### 3.2.2. Synthesis of CR dipeptide compound in the solid phase

For this purpose, CTC (0.8 mmol g^−1^) resin (1.0 g) was washed with DMF and DCM (10.0 mL). Afterwards, *via* DIEA, the first amino acid [Fmoc-Cys(Trt)-OH] (1.0 mmol) was connected to the resin. Next, the capping step was carried out *via* methanol solution; subsequently, the protection group of Fmoc was eliminated with a piperidine solution in the DMF (25%). Next, the mixture of HBTU (1.0 mmol) and DIEA in DMF (5.0 mL) and second amino acid [Fmoc-Arg(pbf)-OH] (0.8 mmol) has been attached. TFA (95%) is used for the last deprotection and separation of the CR dipeptide complex from the CTC. All the process was fulfilled based on the detailed information from the synthesis of the peptide method in our previous works. The structure and purity of the synthesized CR dipeptide have been approved by H-NMR spectroscopy, as given in our previous report.^46^

#### 3.2.3. Conjugation of the β-CD–MPS particles with the synthesized CR dipeptide

In a round-bottom flask (50.0 mL), β-CD–MPS (0.1 g) was dispersed in a solution of H_2_O_2_ (35.0%, 1.0 mL) *via* an ultrasonic ice bath for 10 minutes at 10 °C temperature. After that, the CR dipeptide complex (0.05 g) was added to the flask *via* the drop-by-drop method (dissolved in ethanol). Then, the combination was stirred at 10 °C for 2 h. In the end, the CPFX/β-CD–CR particles were collected by centrifuge (15 min, 4k rpm). Consequently, the particles were dried with a freeze dryer for 48 h.

#### 3.2.4. CPFX loading into β-CD–CR

In this regard, in a glass tube (13 × 100 mm), β-CD–MPS (0.5 g) were dispersed in a provided solution of CPFX (0.1 M) in ethanol (3.0 mL) and DI water (3.0 mL) for 10 min *via* ultrasonic cleaner bath. Next, the combination was stirred in a dark room at 25 °C for 6 h. Finally, the particles were collected by centrifuge (15 min, 4k rpm) and twice rinsed with ethanol to eliminate the adsorbed CPFX over the surfaces. Then the particles were redispersed in deionized water and dried with a freeze dryer for 48 h.

### 3.3. Cellular experiments

#### 3.3.1. Cell cultivation

Initially, the incubation of *S. aureus* and *E. coli* bacterial strains was carried out on Mueller–Hinton agar gel in Petri dishes for 24 h. After that, the 0.5 McFarland solution was prepared using the following process: barium chloride (0.5%) was added to sulfuric acid (0.36 N). The similarity in the opacity of the solution with bacterial cells in the physiological serum with standard solution indicates 1.0–1.5 × 10^8^ CFU mL^−1^ concentration.^67^ These bacteria cells were situated on the agar gel with cotton swabs and incubated at 37.0 ± 1.0 °C with a humidity of 95% for 24 h.

#### 3.3.2. ZOI experiments

First, swabs impregnated the Mueller–Hinton agar gel dishes with bacterial cells. Next, the CPFX, β-CD, CR, and CPFX/β-CD–CR samples were prepared to evaluate the antibacterial activity of the CPFX/β-CD–CR. Then, 5.0 mg of the powder sample was used to put 5.0 mg mL^−1^ of the samples on the agar gel when there were water-undissolved samples. Finally, a cell-impregnated disk was concentratedly placed on the agar gel when there were solution samples. The plate was incubated at 37 °C, with 95% humidity, for 24 h.

#### 3.3.3 .MIC/MBC and colony count experiments

MIC indicates the minimum antibacterial agent required to prevent turbidity due to the bacteria growth in the liquid bacterial culture medium.^68^ The standard Mueller–Hinton broth dilution approach was applied to assess the CPFX/β-CD–CR therapeutic nano bioconjugate antibacterial effect by considering the growth of apparent bacteria in the agar broth. For example, the bacterial strains *S. aureus* and *E. coli* were initially incubated at 37 °C. The inoculated broth-containing control was incubated at 37 °C for 24 h. Next, the samples' inhibition effect was evaluated *via* a modified microdilution approach to clarify their MIC values. Therefore, dilution series in 2.0 mL of Mueller–Hinton broth liquid medium was prepared with pure and 0.039, 0.078, 0.156, 0.3125, 0.625, 1.25, 2.5, 5.0, 10.0, and 20.0 μg mL^−1^ concentrations followed by a bacterial suspension with 0.5 McFarland turbidity. After that, the McFarland standard (10^8^ × 1.5 CFU mL^−1^) was added to the content of 20 μL in each well. Consequently, the microplate was incubated at 37 °C for 24 h, and the MIC results were considered and inquired. MIC referred to the lowest concentration of the CPFX/β-CD–CR therapeutic nano bioconjugate, where no bacterial growth was observed. In addition, MBC is defined as the antibacterial agent's minimum concentration, which reduces the minimum number of bacteria after 24 h to *ca.* 0.001 of the bacteria number at the starting time (*t*_0_). The MBC and MIC determination methods are almost similar; however, the successive dilution procedure was employed for counting the bacteria from the MIC wells concentration upward. The minimum values of MIC and MBC are attributed to the CPFX/β-CD–CR therapeutic nano-bioconjugate onto *S. aureus* and *E. coli* bacteria compared to the individual CPFX. The linear cell culture pattern of *S. aureus* and *E. coli* was made by sterilized inoculation loops on the agar gel, and their colony was spread all over the gel.^69^ Afterward, the sample drop was placed on the disk gel and distributed by an L-shaped glass. This plate was incubated at 37 °C with 95% humidity for 48 h.

### 3.4. Sample preparation for characterization

For electron microscopy, the particles were dispersed in minimum amount of pure ethanol, and ultrasonicated with a cleaner bath (50 kHz, 100 W L^−1^) for 5 min, at an ambient temperature. Afterward, 50 μL of each sample was poured onto a glass laminate, dried in vacuum oven, and then sent to the SEM imaging laboratory. The same procedure was followed for the biological tests, with the difference that the environment in which the whole sample was dispersed in was DMEM instead of ethanol, and the temperature was maintained in a range of 0–5 degrees Celsius (ice bath). The samples were maintained on ice and subjected to living cells in an incubator. For the UV-vis studies, the nanoparticles were dispersed in buffered media, stirred, and finally centrifuged (20 000 rpm, 10 min) to collect the carrier coils. Then, they were filtered with a Whatman filter paper. Also, the blank sample was filtered with Whatman filter paper so as to eliminate the error related to the particulate matter of the paper filter. At last, the solution containing 5.0 mg of CPFX was diluted for a correct UV-vis spectroscopy study.

## 4. Conclusions

We designed a novel DDS which is a complex of nanoscale β-CD with CPFX conjugated to a cell adhesive dipeptide (CR). The nanocomplex's biological activities *versus* Gram-positive (*S. aureus*) and Gram-negative (*E. coli*) bacteria were studied*.* The surface of β-CD was initially modified with MPTMS to introduce –SH groups onto the surface to provide this biocompatible DDS. In addition, the CPFX was loaded to the nanoscale β-CD, and finally, *via* the formation of a disulfide bond, the synthesized CR dipeptide is linked to the surfaces of CPFX/β-CD cargo. This dipeptide sequence was synthesized in the solid phase on CTC resin with high purity. The peptide structures are highly effective, safe techniques and can penetrate cells non-invasive. Also, the usage of this novel-designed strategy prepared a plethora of benefits compared to the usage of free CPFX for bacterial growth prohibition. The primary benefit was using lower doses of antibiotics for similar therapeutic effects. Herein, every essential characterization, including; FT-IR, EDX, TGA, zeta potential, UV-DRS, XRD, FESEM and UV-vis absorbance, was accomplished the received results confirmed with the designed structure.

Furthermore, the drug content and release were investigated with precise analytical methods. The drug loading efficacy was 32%, and the main release was revealed in an acidic environment. Overall, according to the antimicrobial results, it can be deduced that the efficacy of the CPFX is remarkably gained *via* the complexation of nanoscale materials and engineered dipeptides. The growth zone inhibitory of CPFX/β-CD–CR particles on the *E. coli* and the *S. aureus* bacterial cells was 5.5 ± 0.2 cm and 3.5 ± 0.2 cm, respectively. Moreover, the half-life of the provided cargo related to the degradability of the used components in the system ought to be assessed. Following complete degradation, the magnitude of released components' toxicity may be another significant challenge.

## Author contributions

Reza Taheri-Ledari: conceptualization, reviewing, and editing of the main draft, supervision and project administration, and software; Farinaz Jalali: performed all practical sections and also in preparation of the initial draft; Leili Heidari: performed all antimicrobial sections and interpreted the obtained results; Fatemeh Ganjali: wrote the initial draft and interpreted the obtained results; Fereshteh Rasouli Asl: participated in partial parts of the bench work and also in preparation of the initial draft; Simindokht Zarei-Shokat: participated in partial parts of the bench work; Mohadeseh Forouzandeh-Malati: graphics and software; Adibeh Mohammadi: participated in the revision stage; Ali Maleki: managed all sections of the work, supervision, project administration, and financial support.

## Funding

This work was partially supported by the Iran University of Science and Technology.

## Conflicts of interest

The authors declare no competing financial interest.

## Supplementary Material

RA-012-D2RA05822G-s001
