# Supplementary material for: An effective antimicrobial complex of nanoscale β-cyclodextrin and ciprofloxacin conjugated to a cell adhesive dipeptide
Source: RSC Adv. 2022 Dec 15;12(54):35383–95. doi: 10.1039/d2ra05822g (PMC9752432; doi:10.1039/d2ra05822g)
Supplement: RA-012-D2RA05822G-s001 [file RA-012-D2RA05822G-s001.pdf]

Electronic Supplementary Material (ESI) for RSC Advances.
This journal is © The Royal Society of Chemistry 2022

An Effective Antimicrobial Complex of Nanoscale β-cyclodextrin and Ciprofloxacin Conjugated to a Cell Adhesive Dipeptide

Reza Taheri-Ledari[1], Farinaz Jalali[1], Leili Heidari[1,2], Fatemeh Ganjali[1], Fereshteh Rasouli Asl[1], Simindokht Zarei-Shokat[1], Mohadeseh Forouzandeh-Malati[1], Adibeh Mohammadi, Ali Maleki[1,*]

[1] *Catalysts and Organic Synthesis Research Laboratory, Department of Chemistry, Iran University of Science and Technology, Tehran 16846-13114, Iran*

[2] *Department of Chemistry, Faculty of Basic Sciences, Ilam University, P. O. Box 69315-516, Ilam, Iran.*

**Corresponding author. E-mail addresses: maleki@iust.ac.ir (A. Maleki).*

Table of Content

Content	Pages
**Figure S1**. ^{1}H-NMR spectrum of Cys-Arg (CR) dipeptide structure.	S2
**Figure S2**. ^{1}H-NMR spectrum of the neat B-CD.	S3
**Figure S3**. ^{1}H-NMR spectrum of B-CD–MPS.	S4
Interpretations of the NMR spectrum of of B-CD–MPS.	S5
**Figure S4**. ^{1}H-NMR spectrum of CPFX/B-CD–MPS.	S6
**Figure S5.** ^{1}H-NMR spectrum of CPFX/B-CD–MPS (expanded 0.6-4.0 ppm).	S7
**Figure S6.** ^{1}H-NMR spectrum of CPFX/B-CD–MPS (expanded 4.0-9.0 ppm).	S8
**Figure S7.** ^{1}H-NMR spectrum of CPFX/B-CD–CR.	S9
**Figure S8.** ^{1}H-NMR spectrum of CPFX/B-CD–CR (expanded 4.6-9.0 ppm).	S10
**NOTE**	S11

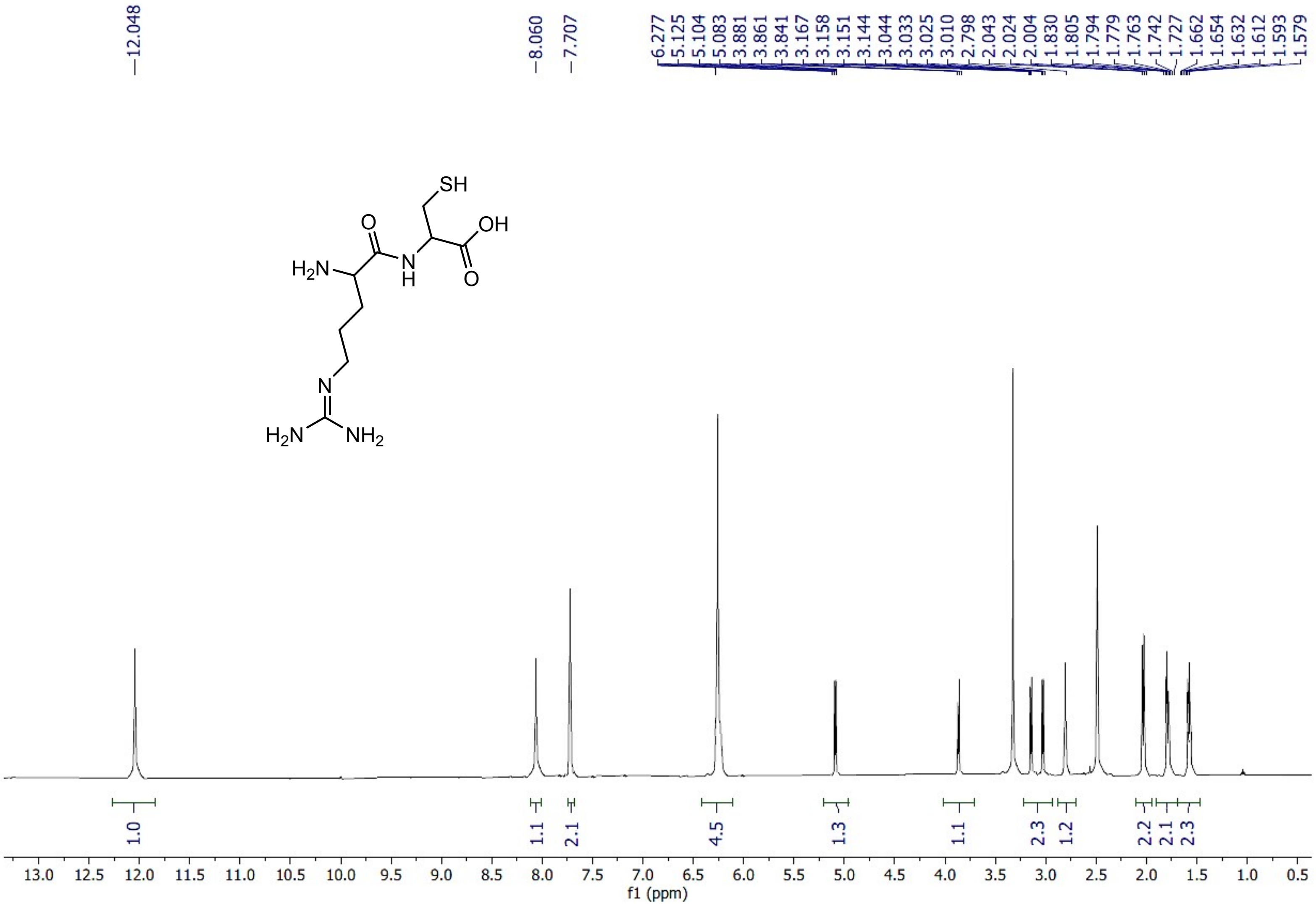

Figure S1. ^{1}H-NMR spectrum of Cys-Arg (CR) dipeptide structure.

Spectral data of CR dipeptide structure:

^{1}H NMR (500 MHz, DMSO): δ = 1.58-1.66 (m, 2H, C*H*$_2$), 1.73-1.83 (m, 2H, C*H*$_2$), 2.02 (t, 2H, *J*= 10 Hz, C*H*$_2$), 2.79 (s, 1H, SH), 3.01-3.17 (m, 2H, CH_2), 3.86 (t, 1H, J = 10 Hz, CH), 5.10 (t, 1H, *J*= 10.5 Hz, CH), 6.28 (s, 4H, NH_2), 7.71 (s, 2H, NH_2), 8.06 (s, 1H, N*H*).

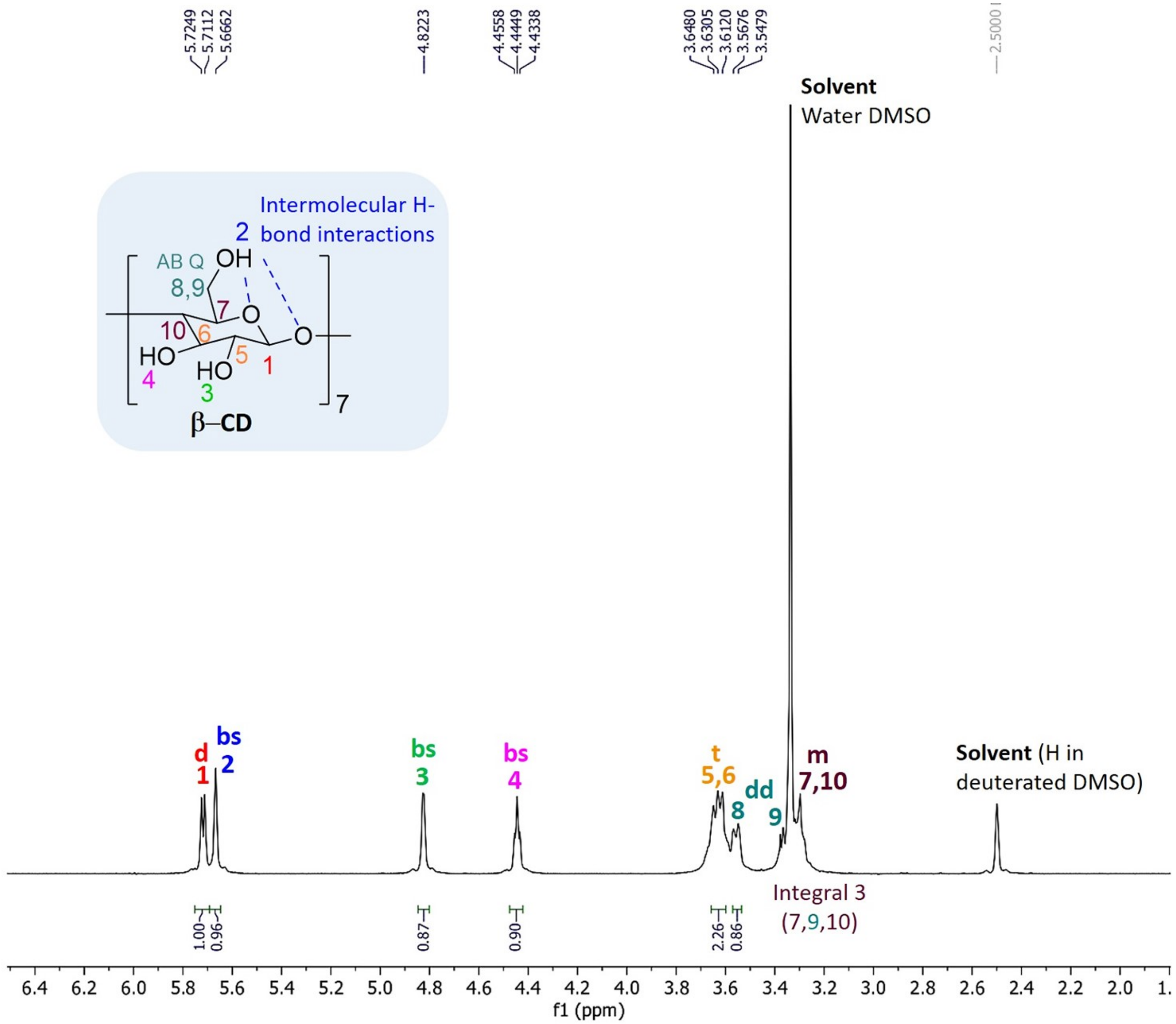

Figure S2. ^{1}H-NMR spectrum of the neat B-CD.

Spectral data of the neat B-CD:

^{1}H NMR (500 MHz, DMSO): δ = 3.25-3.35 (m, 2H, C*H*), 3.37 (dd, 1H, J_1 = 9.8 Hz, J_2 = 31.4 Hz, CH_2OH), 3.55 (dd, 1H, J = 9.8 Hz, J_2 = 31.4 Hz, CH_2OH), 3.63 (t, 2H, C*H*OH), 4.44 (bs, 1H, CHO*H*), 4.82 (bs, 1H, CHO*H*), 5.66 (bs, 1H, CH_2O*H*), 5.71 (d, 1H, J = 6.8 Hz, C*H*O_2).

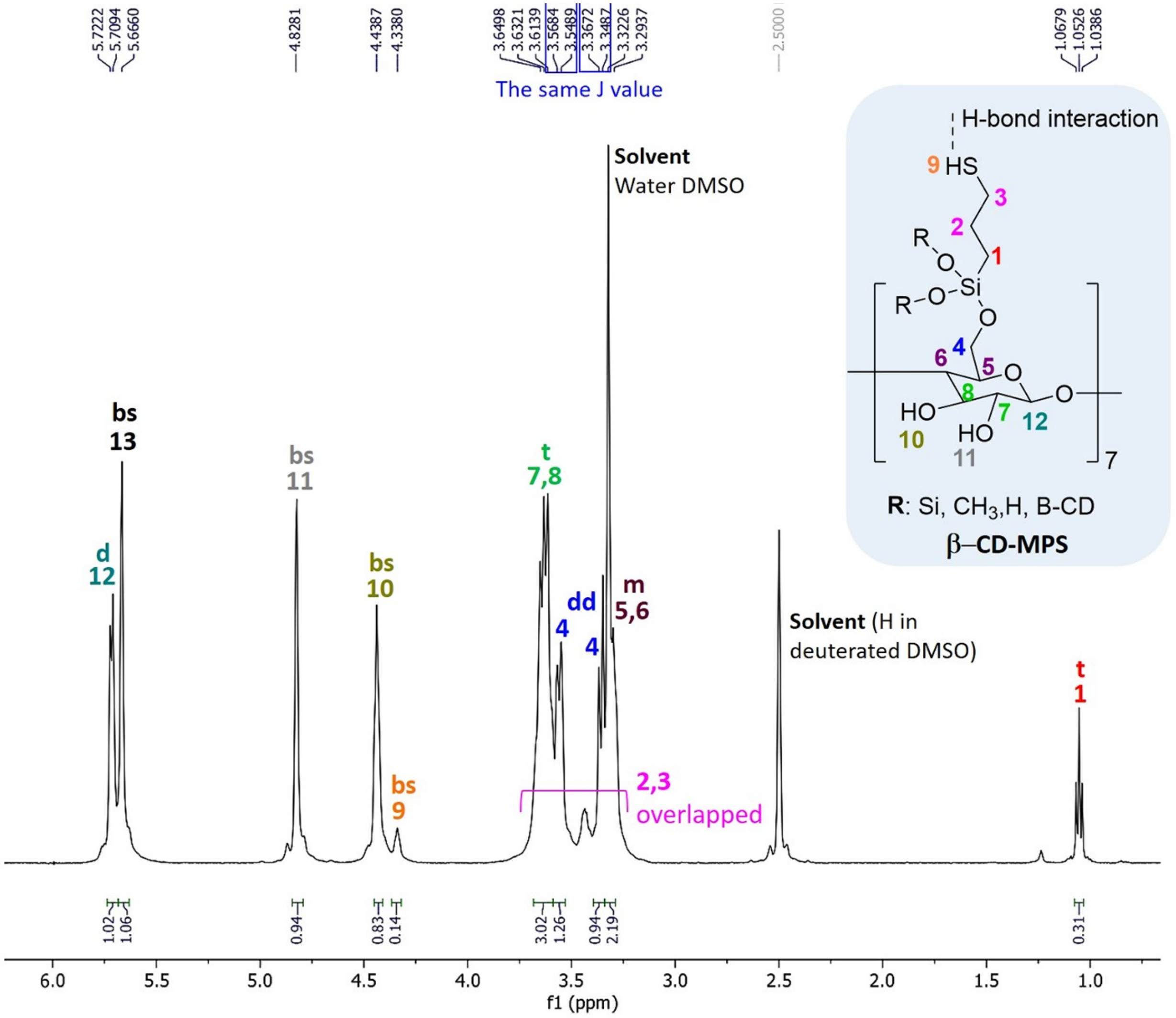

Figure S3. ^{1}H-NMR spectrum of B-CD–MPS.

Spectral data of B-CD–MPS:

^{1}H NMR (500 MHz, DMSO): δ = 1.05 (t, 2H, J = 7.6 Hz, SiCH_2), 3.25-3.32 (m, 14H, CH), 3.35 (dd, 7H, J_1 = 9.2 Hz, J_2 = 100.6 Hz, CH_2OSi), 3.55 (dd, 7H, J_1 = 9.2 Hz, J_2 = 100.6 Hz, CH_2OSi), 3.63 (t, 21H, J = 8.8 Hz, CHOH and SiOH), 4.33 (bs, 1H, SH), 4.43 (bs, 7H, CHOH), 4.82 (bs, 7H, CHOH), 5.66 (bs, 7H, CH$_2$OH), 5.71 (d, 7H, J = 6.4 Hz, CHO$_2$). *All integral values are multiplied by 7.*

Interpretations of the NMR spectrum of of B-CD–MPS:

13

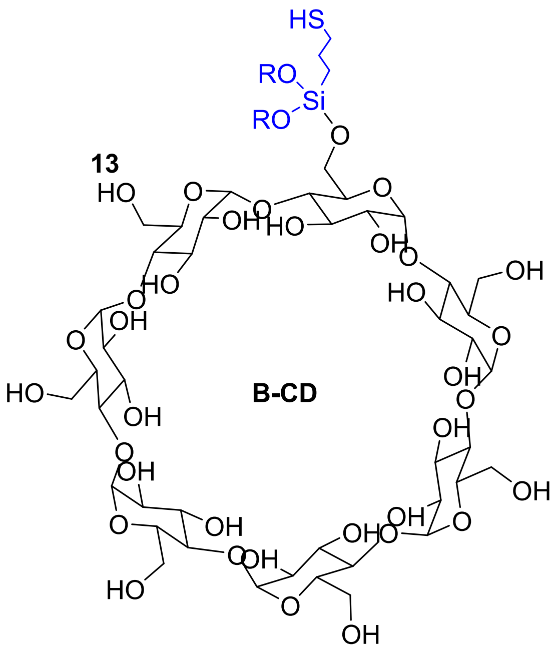

From integral values: It seems that all 7 rings of B-CD are not modified by MPS. In other words, only one ring is modified by MPS. For **1** (methylene next to the Si); 0.31 × 7 ~ 2 protons so, while for other integral values, e.g. proton **12**, 1.02 × 7 ~ 7 protons, which is correct in a 7-ring B-CD monomer. In the same line, for S-H (assigned as **9**); 0.14 × 7 = 0.96 ~ 1 proton. For R–O–Si groups; the presence of Si–O–Si band is confirmed by FTIR (Figure 1b). Also, one proton is excess in the integral 3.02, which can be assigned to the possible formation of SiO–**H**.

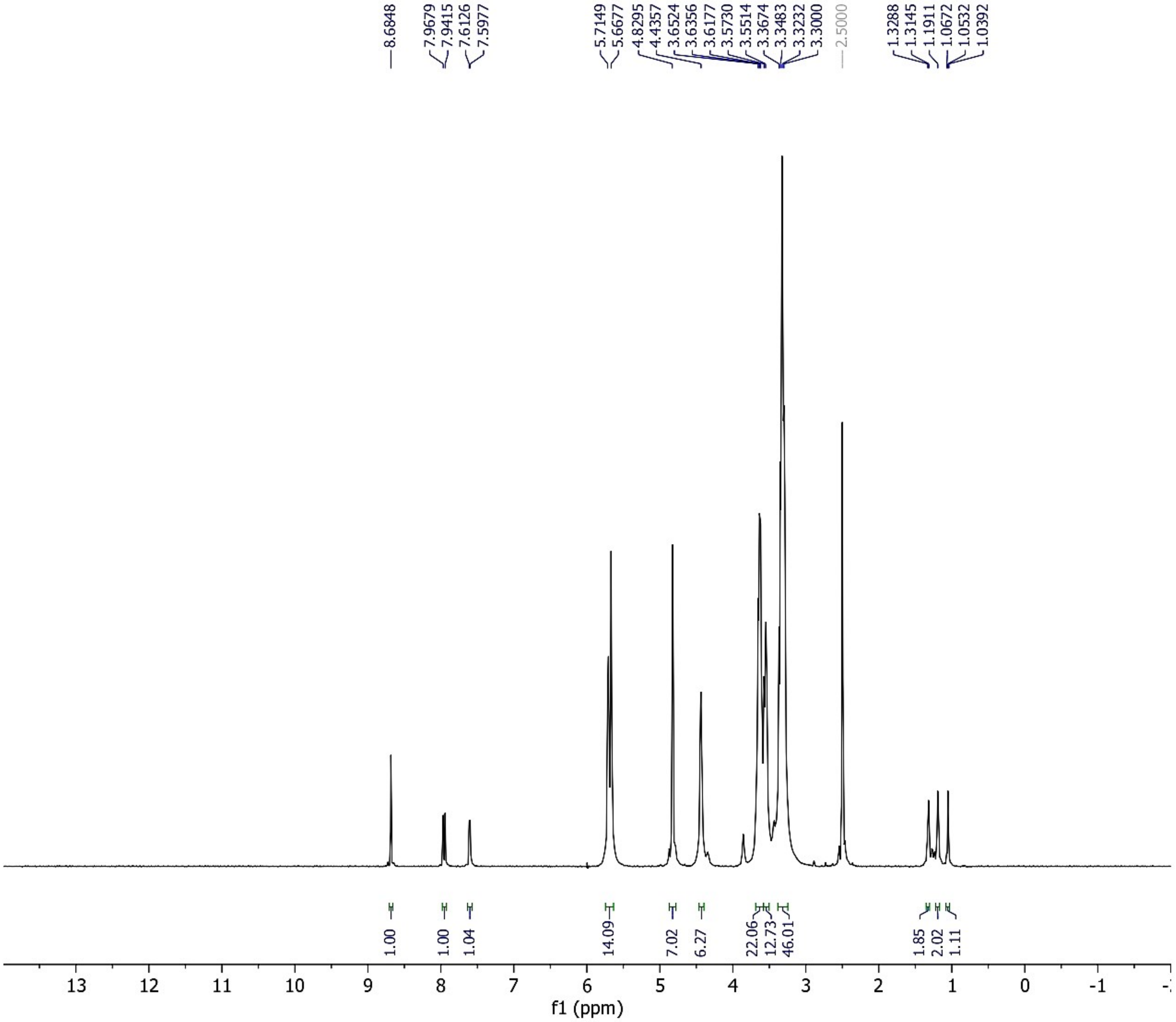

Figure S4. ^{1}H-NMR spectrum of CPFX/B-CD–MPS.

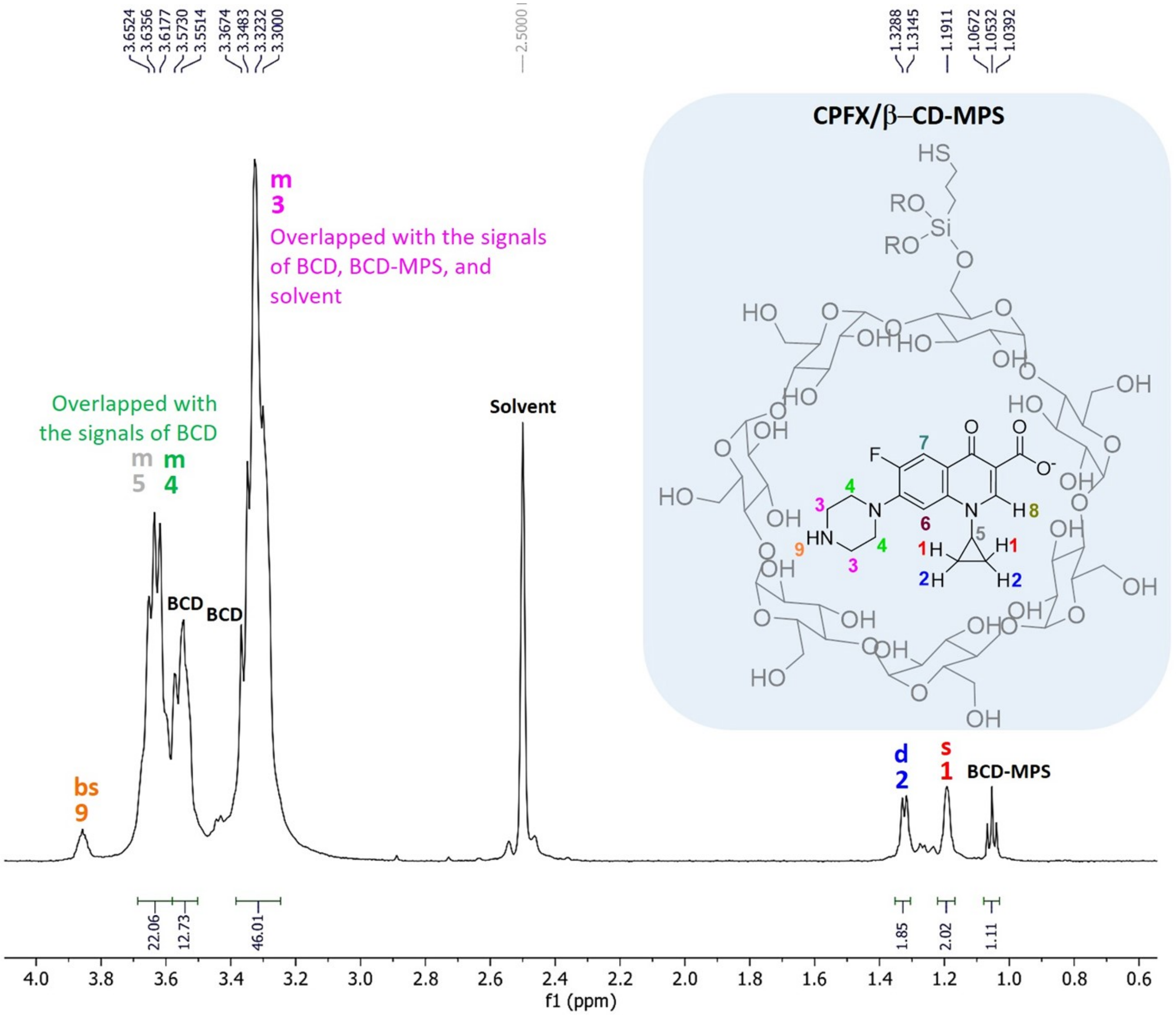

Figure S5. ^{1}H-NMR spectrum of CPFX/B-CD–MPS (expanded 0.6-4.0 ppm).

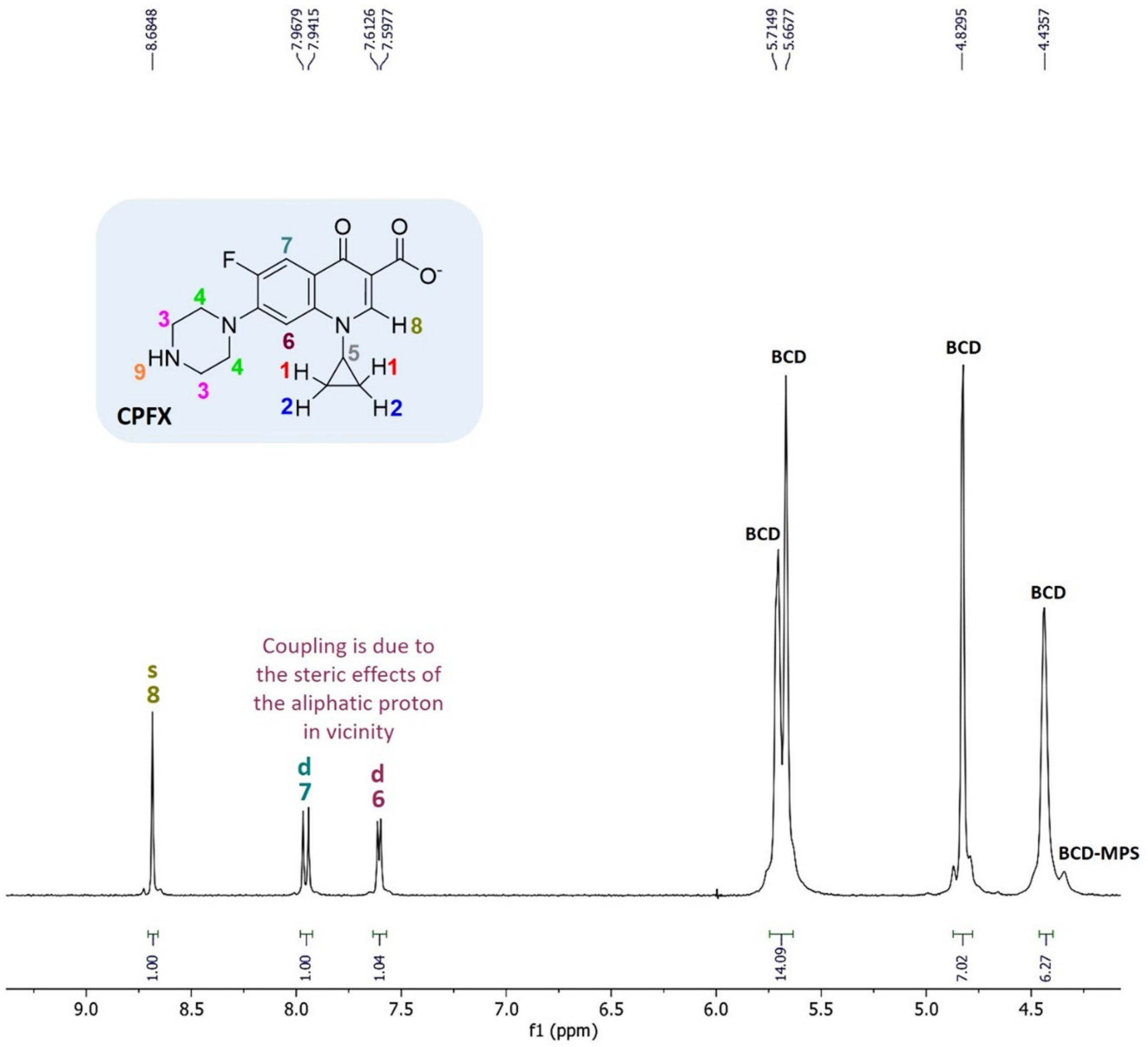

Figure S6. ^{1}H-NMR spectrum of CPFX/B-CD–MPS (expanded 4.0-9.0 ppm).

Spectral data of CPFX/B-CD–MPS:

^{1}H NMR (500 MHz, DMSO): δ = 1.05 (t, 2H, *J* = 7.0 Hz, SiCH_2), 1.19 (s, 2H, CH_2 in CP ring), 1.31 (d, 2H, *J* = 7.0 Hz, CH_2 in CP ring), 3.20-3.70 (m, 81H), 3.85 (bs, 1H, CH_2NHCH_2), 4.30-4.50 (bs, 7H, S*H* and CHO*H* in BCD-MPS), 4.82 (bs, 7H, CHO*H* in BCD-MPS), 5.66 (bs, 7H, CH_2OH in BCD-MPS), 5.71 (d, 7H, CHO_2 in BCD-MPS), 7.60 (d, 1H, *J* = 7.4 Hz, Ar*H*), 7.95 (d, 1H, *J* = 13.2 Hz, Ar*H*), 8.68 (s, 1H, C=C–*H*).

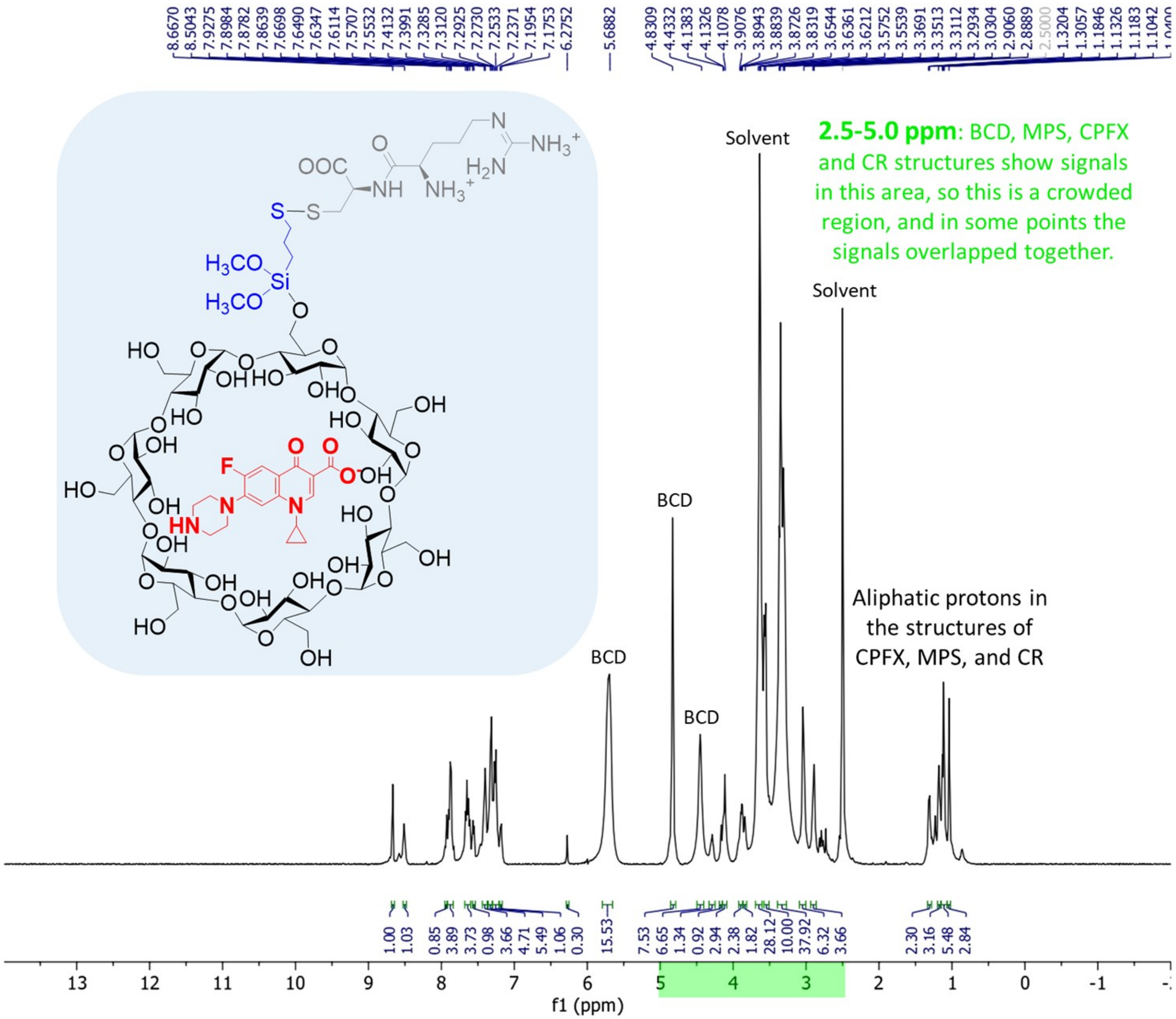

Figure S7. ^{1}H-NMR spectrum of CPFX/B-CD–CR.

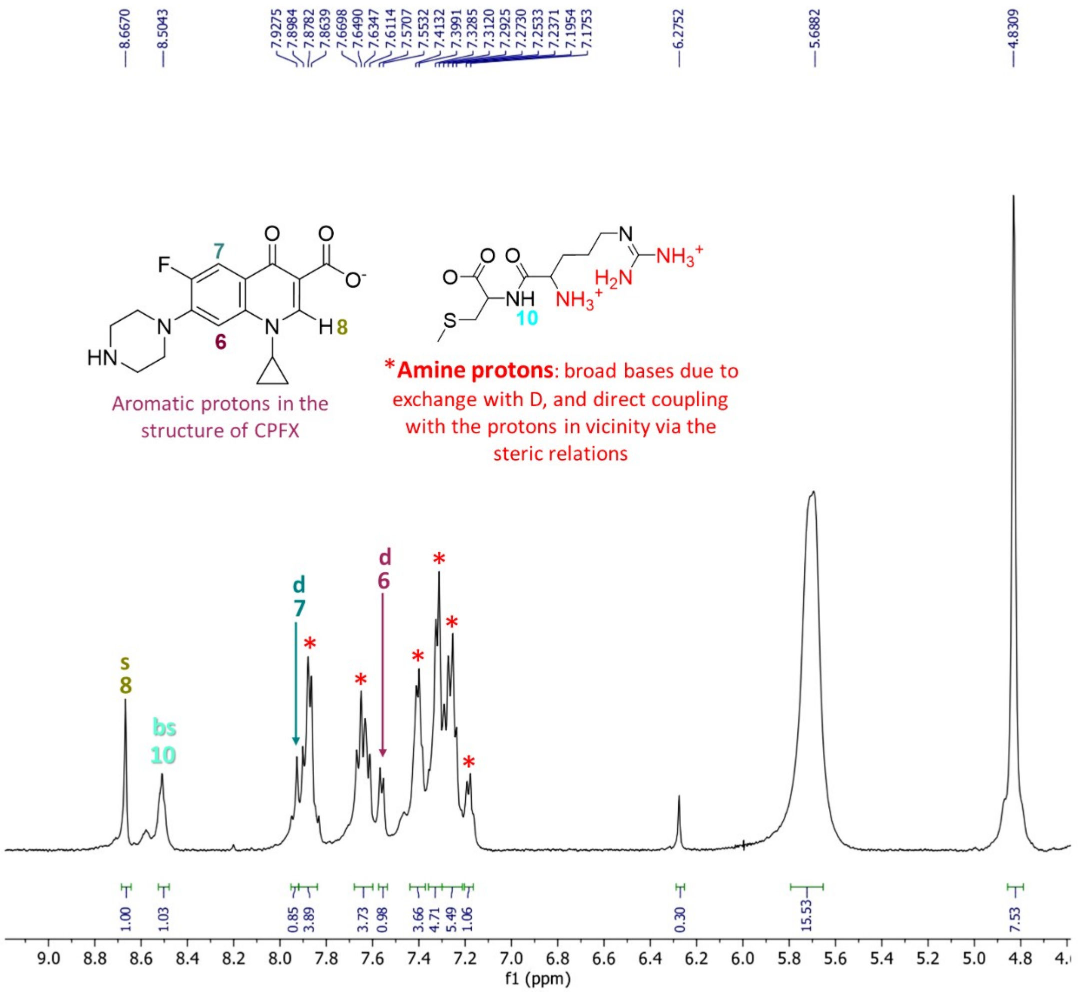

Figure S8. ^{1}H-NMR spectrum of CPFX/B-CD–CR (expanded 4.6-9.0 ppm).

Spectral data of CPFX/B-CD–CR:

^{1}H NMR (500 MHz, DMSO): δ = 0.82-1.32 (m, 13H, Aliphatic H of BCD-MPS, CPFX, and CR), 2.88-4.13 (m, 93H, Aliphatic H of BCD-MPS, CPFX, and CR), 4.43 (s, 7H, BCD), 4.83 (s, 7H, BCD), 5.68 (bs, 15H, BCD), 7.17-7.41 (m, 15H, N*H* in Gn and alpha-amine of R), 7.4 (d, 1H, Ar*H* in CPFX), 7.61-7.66 (q, 4H, *J* = 10 Hz, N*H* in Gn and alpha-amine of R), 7.86-7.89 (d, 4H, *J* = 5 Hz, N*H* in Gn and alpha-amine of R), 7.9 (d, 1H, *J* = 14.5 Hz, Ar*H* in CPFX), 8.5 (bs, 1H, CON*H* in CR), 8.66 (s, 1H, Ar*H* in CPFX).

NOTE: In all H-NMR spectra, there are a few bugs, which have been corrected in the reported spectral data. The mentioned bugs are as below;

1. In spectrum of the neat BCD; the integral value of the signal area of 3.2-3.4 ppm has not been estimated and marked on the spectrum. So, we marked that as integral 3H.
2. In spectrum of CPFX/B-CD–MPS (expanded 0.6-4.0 ppm); the integral value of the first signal (at 1.05 ppm, t) is misestimated, as it should be 2H. Also, a broad-base peak appeared at 3.85 ppm should be considered, as it can be assigned to the N-H (in piperazine ring).
3. In spectrum of CPFX/B-CD–MPS (expanded 4.0-9.0 ppm); the tiny peak appeared at ca. 4.35 ppm should be considered, as it was assigned to the thiol proton (-SH) in the spectrum of B-CD–MPS.
